# P38 MAP Kinase inhibition promotes primary tumour growth via VEGF independent mechanism

**DOI:** 10.1186/1477-7819-7-89

**Published:** 2009-11-15

**Authors:** Adrian W O'Sullivan, Jiang H Wang, Henry P Redmond

**Affiliations:** 1 Department of Academic Surgery, National University of Ireland (NUI) and Cork University College Hospital, Cork, Ireland

## Abstract

**Background:**

The surgical insult induces an inflammatory response that activates P38 MAP kinases and solid tumours can also release cytokines. Therfore inhibition of these pathways may reduce tumour growth We set out to examine the effects of P38-MAPK inhibition on apoptosis, proliferation, VEGF release and cell cycle effects *in-vitro *and on primary tumour growth *in-vivo*.

**Methods:**

4T-1 cells (2 × 10^5^cells/well) were incubated, in 24 well plates with control, 25, 50 or 100 ng/ml of SB-202190 for 24 hours. Cells were subsequently asessed for apoptosis, proliferation, VEGF release and cell cycle analysis. Balb-c mice each received 1 × 10^6 ^4T1 cells subcutaneously in the flank and were then randomised to receive control or SB202190 (2.5 μM/kg) by intraperitoneal injection daily. Tumour size was measured alternate days and at day 24 animals were sacrificed and serum VEGF assessed.

**Results:**

P38-MAPK inhibition *in-vitro *resulted in a significant reduction in proliferation (75.2 ± 8.4% vs. 100 ± 4.3%, p < 0.05) and G_1 _cell cycle phase(35.9 ± 1.1% vs. 32.5 ± 0.6%, p < 0.05) but no significant changes in apoptosis or VEGF levels. *In-vivo*, P38-MAPK inhibition resulted in an increase in primary tumour growth (155.6 ± 34.9 vs. 86.7 ± 18.2 mm^3^, p < 0.05). P38-MAPK inhibition also lowered circulating VEGF levels but this difference was not significant (101.9 ± 27.1 ηg/ml compared to 158.6 ± 27.1 ηg/ml)

**Conclusion:**

These findings demonstrate that P38-MAPK inhibition in-vitro reduces proliferation and G_1 _cell cycle phase as well as promoting primary tumour growth in-vivo. These effects would appear to be independent of VEGF.

## Background

P38 mitogen activated protein kinases (MAPK) are 38-kDa intracellular signal transduction proteins comprising four variants; p38 α, β, γ and δ. Together with c-Jun, amino-terminal kinase and p42/44 MAPK, p38-MAPK forms the MAPK family[1]. MAPK are activated by phosphorylation by MAPK kinases (MKK), as part of intracellular signalling cascades at which diverse extracellular stimuli converge to initiate cellular responses. An important role of MAPK is its activation by a wide variety of stimuli including cytokines, endotoxin, BLP and other stresses, which can ultimately result in the activation of NF-κB[2]. Similarly, as with NF-κB, p38-MAPK has been implicated as a critical mediator of the release of proinflammatory cytokines and positively regulates the expression of a variety of genes involved in the acute phase response such as TNF-α, IL-6 and other inducible enzymes involved in malignant transformation such as VEGF, ERGF and AP-1[3,4]. Expression of proinflammatory cytokines has been reported to promote tumour cell proliferation, host angiogenesis, inflammation and catabolism in animal models and in cancer patients. Elevated levels of pro-inflammatory cytokines have been described in cell line supernatants, tumour specimens and serum of patients with cancer[5,6]. Activation of the MAPK pathway has been shown in the malignant transformation of *in-vitro *cell lines and in *in-vivo *models of colon cancer[7,8]. P38-MAPK activation has been demonstrated in many human cancers but the findings have not been consistent[9]. Some studies have failed to find MAPK activation whereas others have demonstrated NFκB, p38 and JNK activation in colonic polyps[10]. Again as with colon cancer there have been variable reports of p38-MAPK activation in gastric cancer[11]. However, in human non-small cell lung cancer p38-MAPK appears to be constitutively activated and as a result could have an important role in the pathogenesis and progression of certain human cancers[9].

As result p38-MAPK, as a critical mediator of cellular responses, is a suitable candidate as a novel therapeutic strategy for targeting the malignant potential of tumours. Therefore, in the present study we set out to investigate the role of p38-MAPK inhibition using specific p38-MAPK inhibitor (SB-202190) on apoptosis, proliferation, cell cycle and VEGF release *in-vitro *and on tumour growth *in-vivo*.

## Methods

### Reagents

DMEM, PBS, fetal calf serum, penicillin, streptomycin sulphate, and L-glutamine were purchased from Life Technologies (Paisley, Scotland). Propidine iodine (PI), DMSO, PMSF, Nonidet P-40, DTT, HEPES, MgCl2, KCL, NaCl, sodium citrate, Tris, Triton X-100, and EDTA were purchased from Sigma Aldrich (St. Louis, MO). SN50 and RNase were purchased from Calbiochem (San Diego, CA) and Roche (East Sussex, UK), respectively.

**SB-202190**, 1 mg of dry powder was diluted with 3.02 mls of DMSO and maintained as a stock solution of 1 mM at -20°C. 10 μl of this solution was diluted in 10 mls of culture media to obtain a 1 μM working solution and this solution was further diluted with culture media just prior to use to obtain the desired concentrations for the experiment. A control solution was obtained by diluting 10 μl of DMSO in 10 mls of culture media and then further diluting this solution in line with the other concentrations just prior to use.

### Apoptosis Analysis

A murine adenocarcinoma cell line, 4T1 cells (a generous gift from Dr. Fred Miller, Duke University) were maintained as monolayer culture in DMEM supplemented with 10% heat-inactivated foetal calf serum, penicillin (100 units/ml), streptomycin sulphate (100 μg/ml), and L-glutamine (2.0 mM) at 37°C in a humidified 5% CO_2 _atmosphere. Cells (5 × 10^5^cells/well) were incubated, in 6 well plates (Falcon, Lincoln Park, NJ), with control (vehicle-PBS), 25, 50 or 100 μg/ml of SB-202190 for 6 and 24 hours. After the incubation period had finished the suspension of cells was collected in non-adherent FACS tubes (Falcon), and immediately centrifuged at 4°C for 10 minutes at 1,500 rpm. The cell pellet were gently resuspended in 0.5 ml of hypotonic fluorochrome solution (50 μg/ml PI, 3.4 mM sodium citrate, 1 mM Tris, 0.1 mM EDTA, 0.1% Triton X-100) and incubated in the dark before they were analysed on a FACscan flow cytometer (Becton Dickinson, Lincoln Park, NJ). The forward scatter and side scatter of 4T1 cells were simultaneously measured. The PI fluorescence of individual nuclei with an acquisition of fluorescence channel 2 was plotted against forward scatter, and the data was registered on a logarithmic scale. The minimum number of 10,000 events was collected and analysed using the CellQuest software (Becton Dickinson). Apoptotic 4T1 cell nuclei were distinguished by their hypodiploid DNA content from the diploid DNA content of normal 4T1 cell nuclei. 4T1 cell debris was excluded from analysis by raising the forward scatter. All measurements were performed under the same instrument setting.

### Proliferation Analysis

A murine adenocarcinoma cell line, 4T-1 (ATCC, USA) was maintained in culture in full media at 37°C, 5% CO_2 _and 90% humidity. 4T-1 cells (2 × 10^5^cells/well) were incubated, in 24 well plates (Falcon, Lincoln Park, NJ), with control (vehicle-PBS), 25, 50 or 100 ng/ml of SB-202190 for 24 hours. Two hours prior to the end of the incubation period cells were labelled with BrdU (5-bromo-2'-deoxyuridine) a labelling agent provided with a commercially available Cell Proliferation ELISA kit (Roche, Germany). Once the incubation period was finished, the labelling solution was removed by decanting and tapping and cells were further processed and proliferation assessed according to the manufacturers instructions. 200 μl of Fixadent was added to each well and incubated for 30 minutes at room temperature. Flicking and tapping removed the Fixadent solution and 100 μl of the anti-BrdU-POD (peroxidase) working solution was added to each well and incubated for ninety minutes at room temperature. The antibody conjugate was again removed by flicking and tapping. The wells were then washed three times with 200 μl of well washing solution. After washing 100 μl of substrate solution was added to each well and incubated at room temperature for 5-30 minutes until colour development was sufficient for photometric detection. To stop further photometric development 25 μl of 1 M Sulphuric acid was added to each well and mixed thoroughly on a shaker for 1 minute. The optical density of each well was determined within 30 minutes, using a microplate reader set to 450 nm. Control samples were expressed as 100% and treated samples expressed as a relative percentage.

### VEGF Analysis

4T-1 cells (2 × 10^5 ^cells/well) were incubated, in 24 well plates (Falcon), with control (vehicle-PBS), 25, 50 or 100 μg/ml of SB-202190 for 6 and 24 hours. After incubation the suspension of cells were collected and immediately centrifuged at 4°C for 10 minutes at 1,500 rpm. The supernatant was collected and immediately further centrifuged at 4°C for 8 minutes at 10,000 rpm. Supernatant was collected and stored at -80°C for determination of VEGF concentrations using commercially available ELISA kits (R&D Systems, Minneapolis, MN) according to the manufacturer's instructions.

### Cell Cycle Analysis

4T1 cells (1 × 10^6 ^cells/well) were incubated, in 6 well plates, with control (vehicle-PBS), 25, 50 or 100 μg/ml of SB202190 for 24 hours. After the incubation period had finished the suspension of cells were collected in non-adherent FACS tubes and immediately incubated with 0.5 ml of cooled methanol for 30 minutes on ice. After incubation the suspension of cells were centrifuged at 4°C for 5 minutes at 1,500 rpm. The supernatant was discarded and the cell pellet reconstituted with 0.25 ml of 10% propidine iodine solution and 0.25 ml of RNase (200 U/ml). The reconstituted cell suspension was incubated at room temperature for a further 30 minutes in the dark and then filtered through a 35 μm mesh. Cell cycle analysis was performed on a FACscan flow cytometer (Becton Dickinson) with a minimum of 10,000 events collected and analysed using the CellQuest software (Becton Dickinson).

### In vivo animal studies

All animal experiments were carried out in accordance within the Animal Act and under license from the Department of Health and Children, Republic of Ireland. In in-vivo experiments were performed in a blinded fashion. Six to eight week old, male Balb-c mice were supplied by the Biological Services Unit, University College Cork and were allowed free access to food and water in a properly regulated environment throughout the course the experiment. Two hours prior to a flank subcutaneous injection of 0.5 × 10^6 ^of 4T-1 cells suspended in 200 μl PBS, female Balb-c mice (n = 10 per group) were randomised to receive 200 μl of control or 2.5 μM/kg of SB-202190 via an intraperitoneal injection. Tumour size was measured on alternate days and treatment continued on a daily basis for the duration of the experiment. Tumour volumes were calculated using the following formula, V = √(A^2^+B^2^) where A and B are the longest and shortest diameters respectfully.

After 24 days animals were sacrificed and blood collected for cytokine analysis. Blood was collected for cytokine analysis by intracardiac puncture and immediately allowed to stand at 4°C overnight, before centrifugation for 20 minutes at 10,000 rpm. Serum was collected and stored at -80°C for determination of VEGF concentrations using commercially available ELISA kits (R&D Systems) according to the manufacturers' instructions.

### Statistical Analysis

Differences in apoptosis, proliferation, cell cycle and cytokine data compared using one-way analysis of variance with Tukey post-hoc analysis. Inter group differences from in-vivo data were compared using student's t-test. Differences in tumour volumes were compared using a paired t-test. All values were expressed as mean (+/- SEM). P < 0.05 was considered to be statistically significant.

## Results

### In Vitro

**Figure 1:**
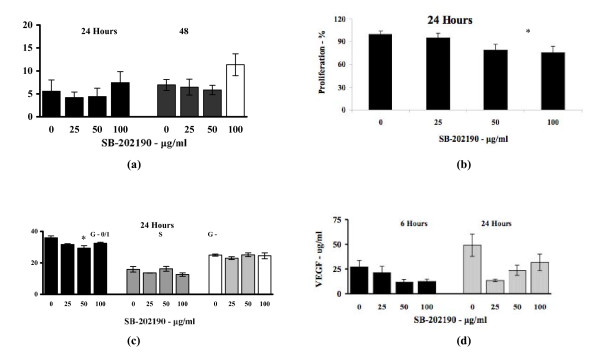
The effect of SB-202190 on 4T-1 cells (a) spontaneous apoptosis (Cells 5 × 10^5 ^were treated with control (vehicle), 25, 50 or 100 μg/ml of SB-202190 for 24 and 48 hours), (b) proliferation (Cells 2 × 10^5 ^were treated with control (vehicle), 25, 50 or 100 μg/ml of SB-202190 for 24 hours, * = p < 0.05), (c) cell cycle analysis (Cells 5 × 10^5 ^were treated with control (vehicle), 25, 50 or 100 μg/ml of SB202190 for 24 hours, * = p < 0.05) and (d) Spontaneous VEGF release (Cells 5 × 10^5 ^were treated with control (vehicle), 25, 50 or 100 μg/ml of SB-202190 for 6 and 24 hours).

**Figure 2:**
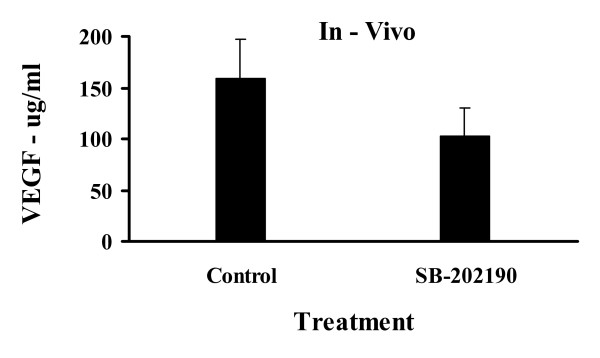
**Serum VEGF levels for control and SB-202190 treated animals**. Animals (n = 10, per group) were randomised to receive control (vehicle) or SB-202190 (2.5 μM/kg) via an intraperitoneal route on a daily basis. After 24 days animals were sacrificed and blood was collected by intracardiac puncture, left to stand overnight prior to centrifugation for 20 minutes at 10,000 rpm. Serum was collected and stored at -80°C for determination of VEGF concentrations using commercially available ELISA kits according to the manufacturer's instructions.

4T-1 cells treated with SB-202190 did not demonstrate significantly increased spontaneous apoptosis compared with control at 24 and 48 hours (7.45 ± 2.4% vs. 7.45 ± 2.4%, p > 0.05) (Fig. 1(a)). However, there did appear to be an increase in apoptosis at both 24 and 48 hours, more marked at 48 hours, but this was only seen with higher doses but was not significant. Cells treated with 100 ng/ml of SB-202190 had significantly reduced proliferation in a dose dependent manner compared to control cells (75.2 ± 8.4% vs. 100 ± 4.3%, p < 0.05) (Fig. 1(b)). Treatment with 25, 50 or 100 ng/ml of SB-202190 had no effect on spontaneous VEGF release at either 6 hours or 24 hours of incubation (p > 0.05) (Fig. 2). Treatment with and 100 ng/ml of SB-202190 led to a significantly greater proportion of cells staying in the G1/G0 resting or pre-replication stage of the cell cycle than compared to control at 24 hours (35.9 ± 1.1% vs. 32.5 ± 0.6%, p < 0.05) (Fig. 1(d)). Treatment with SB-2022190 did not result in any reduction in the number of cells in the S (DNA synthesis) or G2 + M (pre-mitotic and mitotic) phase of the cell cycle (p > 0.05) and a similar pattern was seen at 12 hours.

### In Vivo

**Figure 3:**
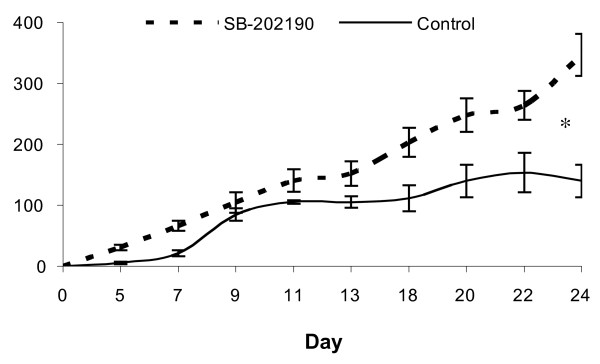
**Tumour volumes over time in response to treatment with control and SB-202190 treated animals**. Animals (n = 10, per group) were randomised to receive control (vehicle) or SB-202190 (2.5 μM/kg) via an intraperitoneal route on a daily basis. (* = p < 0.05).

After 24 days of treatment, the tumour volumes of animals that were treated with SB-202190 were significantly greater than those of control treated animals (155.6 ± 34.9 vs. 86.7 ± 18.2 mm^3^, p < 0.05) (Fig. 3). At the time of sacrifice, although treatment with SB-202190 did result in lower circulating levels of VEGF, this difference was not significant (p > 0.05) (Fig. 2).

## Discussion

P38-MAPK inhibition has been shown to have a survival benefit in a number of *in-vivo *models of sepsis[12]. This survival benefit has been shown to be dependent on a significant reduction in the levels of circulating proinflammatory cytokines as a consequence of down regulation of p38 MAPK *in-vivo*. Many tumour cell lines, tumour specimens and serum of patients with cancer have been shown to have elevated levels of proinflammatroy cytokines[13,14]. Proinflammatory cytokines have also been reported to promote cell proliferation and have been associated with increased potential for tumour growth[15]. Given that human non-small cell lung carcinoma demonstrates selective activation of p38-MAPK, it would provide a mechanism of targeting malignant cell growth or transformation. *In-vitro *p38-MAPK inhibition did show some potential as a possible strategy with a trend of increasing apoptosis and reduced proliferation. It also had some reduction in VEGF release and beneficial effects on cell cycle kinetics. However, *in-vivo *p38-MAPK inhibition led in a significant increase in primary tumour growth, which was independent of VEGF. We have demonstrated similar effects with NFκB inhibition; increased primary tumour growth independent of VEGF with down regulation of NFκB in tumour extract (unpublished data). Although the effect of p38-MAPK inhibition in animal models of sepsis and human endotoxaemia has been shown to be beneficial, our results show that any anti-tumourgenic trends seen *in-vitro *do not translate to a beneficial effect *in-vivo*. The role of p38 MAPK in human cancers is unclear and confusing[10,16]. Some reports have shown p38-MAPK activation in human cancers whereas others have not demonstrated such activation. In other studies the pattern of p38-MAPK activation has been inconsistent and equivocal. Our study showed that although p38-MAPK inhibition appeared to have beneficial effects in terms of malignant potential on a cell line in vitro, this effect was not translated to the same cell line *in-vivo*. This may well be due to a dampening of the inflammatory response and the necessary host inflammation to recognise the tumour as non self, but may also relate to the wide variety of genes P38-MAPK can activate. Although, P38-MAPK may be a critical mediator of the inflammatory response its role in tumour growth is relatively unknown. Indeed many studies suggest that the role of p38-MAPK cascade in human cancers may be cell or tissue specific which would make its effect unpredictable and indeed this effect may vary between the same tumour types[17]. This effect in the current study does not appear to be mediated via VEGF but it may be difficult to identify a particular mediator. This may be due to the variation of P38-MAPK activation in human tumours and the diverse range of intracellular mediators mediated via p38 MAPK. P38-MAPK inhibition can have some effects *in-vitro *but this effect has not translated into benefit in an *in-vivo *setting. Although *in-vivo *this effect does not appear to be mediated via VEGF, further studies are needed to identify the pathways involved following p38-MAPK activation or inhibition.

## Competing interests

The authors declare that they have no competing interests.

## Authors' contributions

AOS carried out the in-vitro and in-vivo experimetnal work and drafted the manuscript. JHW participated in the design of the study and performed the statistical analysis. HPR conceived of the study, and participated in its design and coordination. All authors read and approved the final manuscript.
